# New avenues in the medical treatment of Cushing’s disease: corticotroph tumor targeted therapy

**DOI:** 10.1007/s11060-013-1151-1

**Published:** 2013-05-15

**Authors:** Maria Fleseriu, Stephan Petersenn

**Affiliations:** 1 Departments of Medicine and Neurological Surgery, and Northwest Pituitary Center, Oregon Health & Science University, Mail Code BTE472, 3181 S. W. Sam Jackson Park Road, Portland, OR 97239 USA; 2 ENDOC Center for Endocrine Tumors, Altonaer Str. 59, 20357 Hamburg, Germany

**Keywords:** Cushing’s disease, Pituitary corticotroph adenoma, Somatostatin analog, Cabergoline, Pasireotide, Retinoic acid

## Abstract

Cushing’s disease (CD) is a condition of chronic hypercortisolism caused by an adrenocorticotropic hormone-secreting pituitary adenoma. First-line transsphenoidal surgery is not always curative and disease sometimes recurs. Radiotherapy often requires months or years to be effective, and is also not curative in many cases. Consequently, effective medical therapies for patients with CD are needed. Corticotroph adenomas frequently express both dopamine (D2) and somatostatin receptors (predominantly sstr_5_). Pasireotide, a somatostatin analog with high sstr_5_ binding affinity, has shown urinary free cortisol (UFC) reductions in most patients with CD in a large phase 3 trial, with UFC normalization and tumor shrinkage in a subset of patients. Adverse events were similar to other somatostatin analogs, with the exception of the degree and severity of hyperglycemia. Two small trials (one prospective and one retrospective) have suggested that cabergoline, a D2 receptor agonist, could be effective in normalizing UFC, but current long-term data results are conflicting. Combination treatment with pasireotide plus cabergoline and the adrenal steroidogenesis inhibitor ketoconazole has been successful, but further investigation in larger trials is necessary. Retinoic acid also showed interesting results in a recent very small prospective study. Glucocorticoid receptor blockade with mifepristone has recently demonstrated improvement in signs and symptoms of Cushing’s and glycemic control; however, this modality does not address the etiology of the disease and has inherent adverse events related to its mechanism of action. Pituitary-targeted medical therapies will soon play a more prominent role in treating CD, and may potentially become first-line medical therapy when surgery fails or is contraindicated.

## Introduction

Cushing’s disease (CD) is a condition of hypercortisolism caused by an adrenocorticotropic hormone (ACTH)-secreting pituitary adenoma. While rare, it is associated with significant morbidity and mortality [1–3].

**Fig. 1:**
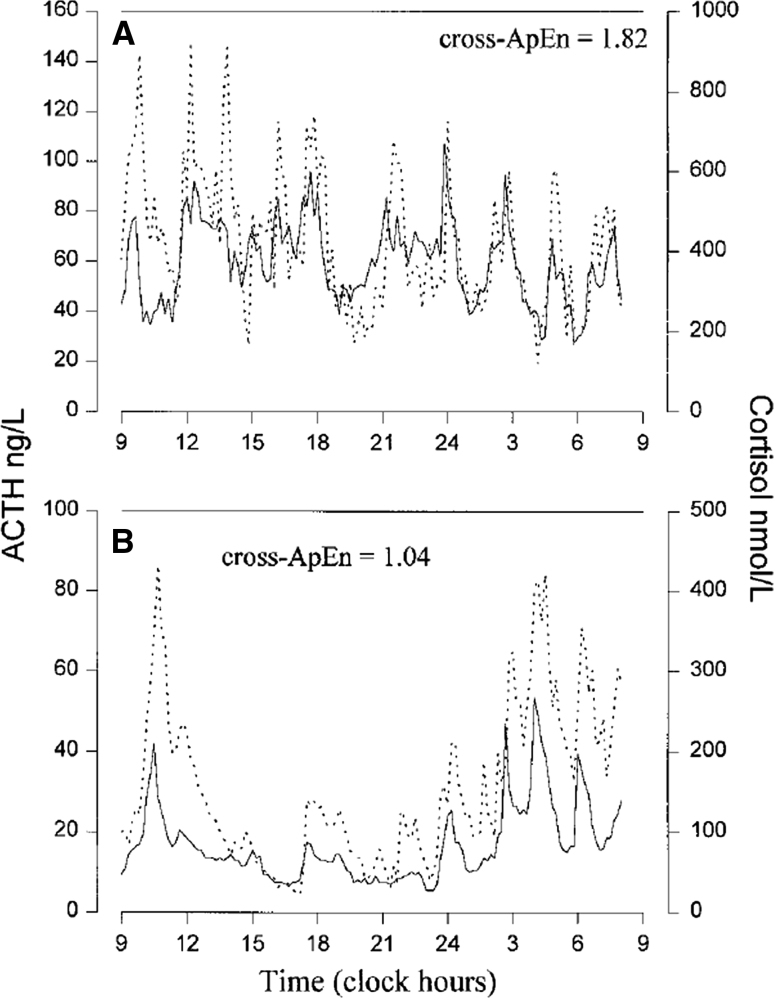
24-hour plasma levels of ACTH (*dotted line*) and cortisol (*solid line*) in a normal female (**a**) and a female patient with Cushing’s disease (**b**). Note that in CD, both basal and peak cortisol levels are elevated, and cortisol secretion is less synchronized to ACTH levels. From Roelfsema F, Pincus SM, Veldhuis JD (1998) *J Clin Endocrinol Metab,* volume 83, page 690 [7]. Reproduced with permission

Pituitary secretion of ACTH is normally regulated by hypothalamic and paracrine signals, and through negative feedback by glucocorticoids [4]. Systemic ACTH levels also exhibit a circadian rhythm, controlled by the suprachiasmatic nucleus and entrained by light–dark cycles [4]. Superimposed on this circadian rhythm is an ultradian cycle of pulsatile ACTH release, which is closely mimicked by cortisol levels (Fig. 1a) [5–7]. In patients with CD, both basal and peak ACTH levels are elevated (Fig. 1b), and ACTH and cortisol levels are uncoordinated [5–10].

For most patients with CD, primary treatment is transsphenoidal surgery to remove the pituitary adenoma. However, success rates are variable (reported as 65 to 90 %) and dependent on the surgeon’s expertise [11]. Published 5- and 10-year recurrence rates are as high as 25 and 56 %, respectively [12, 13], and many patients develop deficiencies of other pituitary hormones post-surgery [11]. Patients who fail to achieve or maintain remission require secondary treatment, including radiotherapy and/or adrenalectomy—both of which are associated with subsequent hormone deficiencies [11]. Thus, additional treatment options for patients with CD are required.

While there are several potential CD therapeutic targets, there is relatively little clinical experience with most medical treatments. Recently, however, prospective studies have demonstrated promise for pituitary-directed medical interventions targeting the underlying adenoma.

## Potential pituitary targets and pituitary-targeted therapies for corticotroph adenomas

### Somatostatin receptors in the pituitary and pituitary adenomas

Somatostatin often serves to inhibit secretory responses, but also exhibits antiproliferative effects in some tissues [14]. Somatostatin receptors are widespread throughout the central nervous system and in several peripheral tissues [15, 16]. To date, 5 somatostatin receptor subtypes (sstr) have been identified and cloned (sstr_1_–sstr_5_) [14]. Immunostaining indicates that normal human anterior pituitary expresses all 5 subtypes, and that most corticotroph adenomas express ≥1 sstr [17–19]. While expression levels are highly variable, sstr_2_ and sstr_5_ are most consistently expressed, and sstr_4_ is generally absent [17, 19]. A study using quantitative RT-PCR and immunohistochemistry found that human corticotroph adenomas expressed sstr_1,2,4 and 5_, and that sstr_5_ had the highest expression levels in 10/12 adenomas [20].

Differential sstr expression levels between somatotroph and corticotroph adenomas may partly explain why octreotide, which has relative selectivity for sstr_2_, inhibits growth hormone secretion from somatotroph adenomas but has little effect on ACTH secretion from corticotroph adenomas [19]. Somatostatin has also been shown to inhibit ACTH secretion from pituitary cells taken from adrenalectomized rats [21], and reduced serum ACTH levels in humans who had hypocortisolism [22–24], but did not affect ACTH secretion from pituitary cells taken from normal rats [25, 26] or ACTH levels in patients with CD [17, 19, 27–29]. These findings suggested that high corticosteroid levels were associated with low responsiveness to sstr_2_-specific analogs in corticotroph adenomas. This conclusion is supported by the observation that the ability of somatostatin or octreotide to inhibit ACTH secretion from cultured corticotroph adenoma cells was abolished by pretreatment with dexamethasone [30].

These findings have produced a working model of the regulation of ACTH secretion by corticotroph adenomas [31, 32], wherein high systemic cortisol downregulates sstr_2_ expression in the corticotroph adenoma cells, rendering sstr_2_ agonists ineffective at inhibiting ACTH secretion. In contrast, sstr_5_ expression appears to be relatively unaffected by high cortisol levels. Upon treatment with an sstr_5_ agonist, ACTH secretion is reduced, leading to declines in cortisol secretion from the adrenal glands. This model is supported by evidence from murine AtT20 cells, a corticotroph cell line. Treatment of AtT20 cells with dexamethasone reduced expression of sstr_2_ mRNA without significantly affecting sstr_5_ mRNA expression or the ability of an sstr_5_ agonist to inhibit ACTH secretion [33].

## Somatostatin analogs

### Overview

**Fig. 2:**
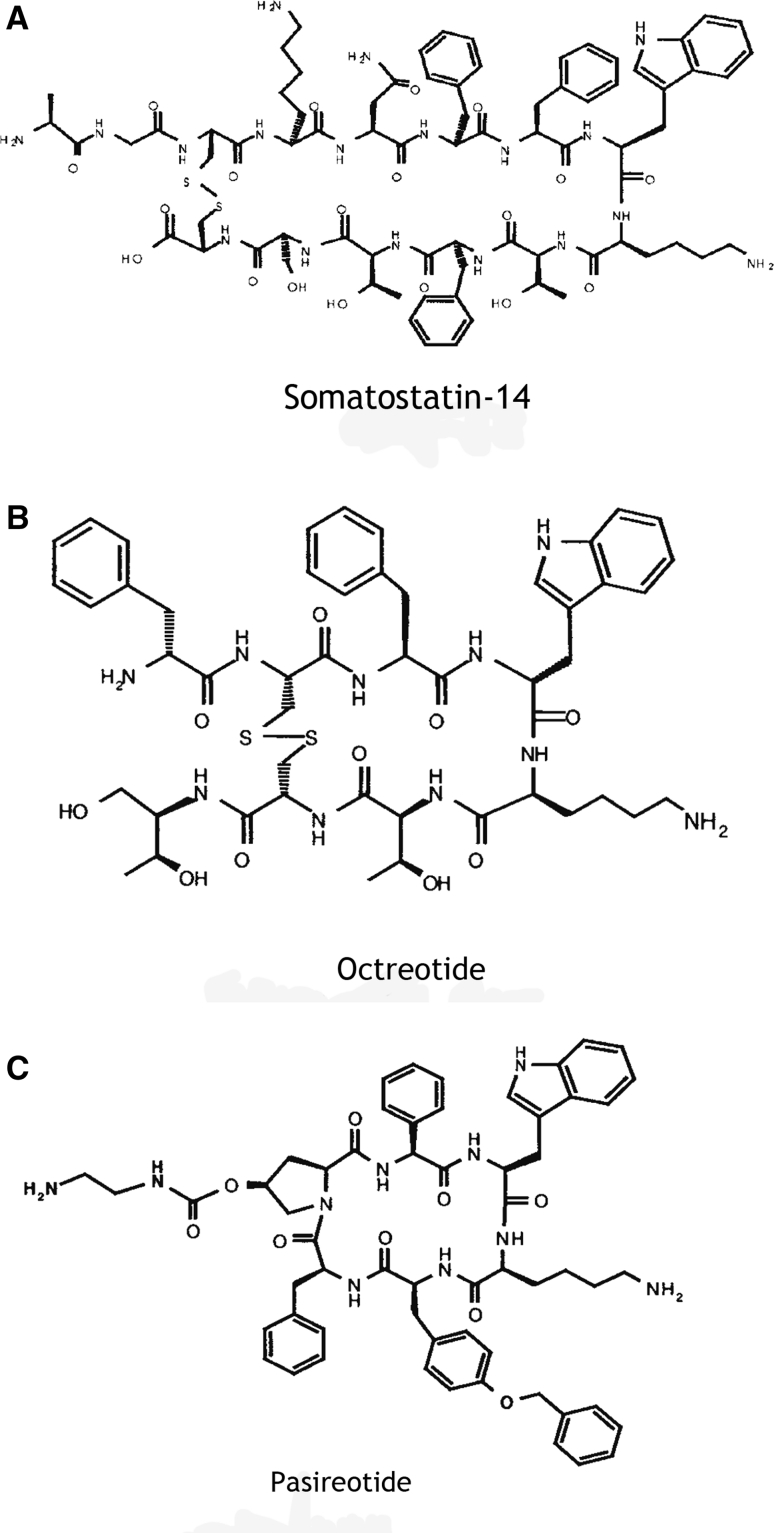
Chemical structures of somatostatin-14, octreotide, and pasireotide. Adapted from Bruns C, Lewis I, Briner U, Meno-Tetang G, Weckbecker G (2002) ©*European Journal of Endocrinology*, volume 146, page 710 [38]. Reproduced with permission

Generally, somatostatin analogs are designed to emulate the structure of native somatostatins, while lacking the enzyme degradation sites of the native molecules. Octreotide and lanreotide (Fig. 2a, b) have long been used for treatment of acromegaly, hyperthyroidism, and gastroenteropancreatic neuroendocrine tumors. Another novel analog, somatoprim (DG3173), selectively binds sstr subtypes 2, 4, and 5 and has demonstrated suppression of growth hormone in octreotide-non-responsive cultured human somatotroph adenomas [34]. However, the clinical utility of somatoprim has not yet been evaluated.

Table 1 [35–38] summarizes binding affinities (IC_50_) for somatostatin-14 and for 5 somatostatin analogs. Somatastain-14 binds to all 5 sstrs with similar binding affinity, while both octreotide and lanreotide exhibit relative selectivity for sstr_2_. This selectivity profile is consistent with studies that have ascribed the efficacy of octreotide and lanreotide at inhibiting growth hormone release from somatotroph adenomas to their activity at sstr_2_ [39].

**Table 1:** Binding affinities (IC_50_) for various somatostatin analogs at the 5 known somatostatin receptor subtypes

	Binding affinity (IC_50_), nmol/L				
Analog	sstr_1_	sstr_2_	sstr_3_	sstr_4_	sstr_5_
Somatostatin-14	0.93	0.15	0.56	1.5	0.29
Octreotide	280	0.38	7.1	>1000	6.3
Lanreotide	180	0.54	14	230	17
Vapreotide	>1000	5.4	31	45	0.7
Pasireotide	9.3	1.0	1.5	>100	0.16
Somatoprim [35]	>1000	3	>100	7	6

## Pasireotide

### Overview

Pasireotide is a cyclic hexapeptide that was discovered using a rational approach to design a stable somatostatin analog with a receptor selectivity profile resembling that of native somatostatin. Pasireotide (Fig. 2c), has high affinity for sstr_1,2,3 and 5_ (Table 1), with much higher affinity for subtypes 1 and 5, and lower affinity for subtype 2, compared with octreotide [40]. In preclinical studies, pasireotide demonstrated inhibition of ACTH secretion in cultured human corticotroph adenomas [19, 20] and in murine AtT20 cells [17, 19]; one study has also reported reduced cell proliferation in cultured human corticotropinomas [20]. Compared with octreotide, pasireotide demonstrated greater suppression of CRH-stimulated ACTH release from AtT20cells [17, 19]. Further, consistent with the model of glucocorticoid-mediated downregulation of sstr_2_, pretreatment of AtT20 cells with dexamethasone abolished the inhibitory effects of octreotide on ACTH secretion, but had no effect on the inhibitory action of pasireotide or somatostatin-14 [33]. Together, these findings support the conclusion that pasireotide inhibits ACTH secretion from corticotroph adenomas by acting predominantly at sstr_5_. Subsequent supportive in vivo results [41, 42] established the rationale for studies on the use of pasireotide in patients with CD.

### Clinical trial experience with pasireotide in Cushing’s disease

After promising results in a 15-day Phase 2 trial [40], a randomized, double-blind phase 3 trial of pasireotide was conducted in adult patients with de novo, persistent, or recurrent CD (UFC ≥1.5× upper limit of normal [ULN]) who were not candidates for surgery and with no pituitary radiotherapy in the preceding 10 years. Eligible patients (*N* = 162) were randomized to receive 600 or 900 mcg of pasireotide twice daily for an initial period of 3 months. After 3 months, those with UFC <baseline and <2× ULN continued blinded therapy for another 3 months. Other patients were unblinded, and their dose was increased by 300 mcg twice daily. At 6 months, all patients entered the open-label phase of the study and could have their dose increased again to a maximum of 1200 mcg twice daily, if necessary [43].

**Fig. 3:**
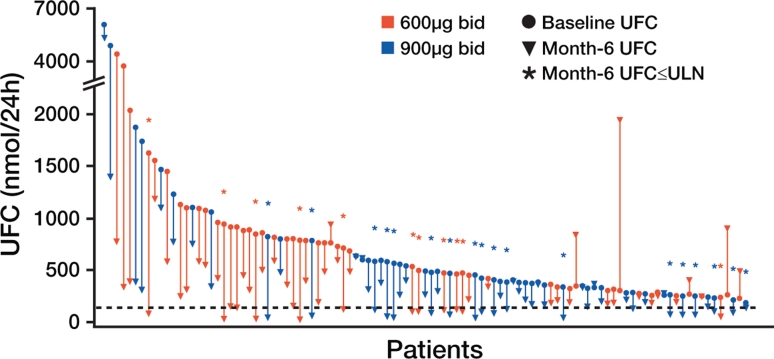
Changes in UFC levels from baseline to 6 months in individual patients in the phase 3 trial of pasireotide. The dashed black line represents the upper limit of the normal range for UFC. From Colao A, Petersenn S, Newell-Price J, Findling JW, Gu F, Maldonado M, et al., for the Pasireotide B2305 Study Group (2012) *N Engl J Med*, volume 366, page 918 [43]. Reproduced with permission

Figure 3 shows changes from baseline to 6 months in UFC levels for patients in the two dose groups of the phase 3 trial. The majority of patients had declines in UFC levels at 6 months; 6 patients had increases. At 6 months, 15 % of patients in the 600 mcg group and 26 % of patients in the 900 mcg group met the primary endpoint of UFC level within the normal range without dose increase. Median percentage changes in UFC levels from baseline to 6 months were −47.9 % in the 600 mcg group and −47.9 % in the 900 mcg group. Among the 36 patients who achieved normalization of UFC levels at 6 months, 20 maintained normal levels at 12 months, including some patients who had dose reductions. Moreover, patients who responded to treatment could generally be identified within the first 2 months of treatment [43].

Mean plasma ACTH levels and serum and salivary cortisol levels were reduced at both 6 and 12 months, and patients had significant improvements in signs and symptoms of hypercortisolism during the trial, including reductions in systolic and diastolic blood pressure, triglycerides, low-density lipoprotein cholesterol, and body weight. The time-course of many of these improvements closely followed the time-course UFC reduction. Patients also reported significant increases in health-related quality of life. Finally, among the 75 patients who had measurable pituitary tumors on magnetic resonance images at baseline, tumor volume changed by an average of −9.1 and −43.8 % in the 600 and 900 mcg groups, respectively [43].

Hyperglycemia-related adverse events occurred in 118 of 162 patients (73 %), and 10 patients discontinued because of such events. Twenty one patients (13 %) had grade 3 or 4 hyperglycemia, and 74 patients initiated a new antidiabetic medication during the study [43]. While no glycemic intervention studies have been completed in pasireotide-treated CD patients, the mechanism of pasireotide-induced hyperglycemia was investigated in 90 healthy volunteers [44]. Results indicate that pasireotide-induced hyperglycemia is mediated by a reduction in secretion of insulin and incretin hormones glucagon-like peptide-1 (GLP-1) and gastric inhibitory polypeptide (GIP). Glucagon secretion was only mildly inhibited, and pasireotide appears to have no effect on peripheral or hepatic insulin sensitivity in healthy individuals. Treatment with the GLP-1 analog liraglutide or the dipeptidyl peptidase 4 (DPP-4) inhibitor vildagliptin was most effective in countering hyperglycemia in this population, while metformin and nateglinide had little effect.

Patients with CD, however, are often insulin-resistant. A recently published proposal for management of hyperglycemia in patients with CD treated with pasireotide recommends metformin as first-line medical treatment for CD patients who develop new or worsening hyperglycemia with pasireotide, with an adjunctive DPP-4 inhibitor, sulfonylurea/glinide, and/or GLP-1 analog as required to achieve glycemic control. Metformin plus insulin is recommended if such combination therapy is not tolerated or insufficient and early intervention with insulin is recommended on a case-by-case basis [45].

Other than the degree and severity of hyperglycemia-related events, the safety and adverse event profiles of pasireotide in the phase 3 trial were similar to those observed with other somatostatin analogs. Other common adverse events were diarrhea (58 %), nausea (52 %), cholelithiasis (30 %), headache (28 %), and abdominal pain (24 %). Among the 137 patients who had a normal gallbladder at baseline, 27 had gallstones at their latest assessment and 6 had a cholecystectomy [43]. Pasireotide was recently approved in the US [46] and Europe [47] for the treatment of adult CD patients for whom pituitary surgery is not an option or has failed.

### Dopamine receptors and dopamine receptor agonists

Like somatostatin receptors, dopamine receptors are widely expressed in normal neuro-endocrine tissues and pituitary adenomas, including approximately 80 % of corticotroph adenomas [48, 49]. Five dopamine receptor subtypes have been characterized, usually classified as D_1_-like or D_2_-like, with most D_2_-like receptors associated with inhibitory actions [50]. The dopamine agonists bromocriptine and cabergoline have both been used to treat pituitary adenomas; however, cabergoline is more selective at D_2_ receptors and has been found to be more effective and better tolerated than bromocriptine in women receiving treatment for hyperprolactinemia [51].

In clinical studies, bromocriptine has had small and variable effects on ACTH secretion in patients with CD [50]. In contrast, a 2004 study demonstrated significant in vitro inhibition of ACTH secretion with cabergoline in 100 % of cases with confirmed D_2_ receptor expression, and significant in vivo reduction of cortisol levels in 60 % of cases—with 40 % normalization of cortisol secretion [48]. Altogether, 3 small, non-randomized studies (one with a long-term follow-up publication) have found evidence have found evidence for efficacy of cabergoline in this setting [48, 52, 53].

In a study of 10 patients, 6 achieved UFC normalization after 3 months’ treatment with cabergoline, and 2 additional patients achieved significant UFC reductions [48]. In a follow-up to this investigation, 20 patients were enrolled who had CD unsuccessfully treated by surgery [53]. After 3 months of cabergoline (1 mg/week with dose adjusted to a maximum of 7 mg/week), 7 patients (35 %) achieved eucortisolism and an additional 8 (40 %) experienced ≥25 % decline in UFC. The 8 partial responders all had dose increases, and 6 of these had normal UFC after 6–12 months. However, 5 patients who achieved normal UFC levels either initially or after dose increase escaped from treatment control. Thus, at 12 months, 10 patients (50 %) had normal UFC levels. However, 2 of these patients subsequently discontinued treatment because of severe asthenia, resulting in a long-term control rate of 40 %. Most responders exhibited improvements in signs and symptoms of CD, including significant mean declines in blood pressure, waist/hip ratio, serum glucose, and tumor volume, and significant improvements in β-cell function.

Similar supportive results were found in a retrospective, non-randomized analysis of 30 patients with CD in Buenos Ares and Montreal who received cabergoline (initially 0.5–1.0 mg/week adjusted to a maximum of 6 mg/week) for up to 5 years [54]. Within 3–6 months, 11 patients (37 %) achieved sustained normalization of UFC (≥2 normal values measured at 1–3 months apart; complete response). Nine of these patients achieved long-term UFC normalization after a mean of 37 months (range, 12–60) on a mean cabergoline dose of 2.1 mg/week. Four patients (13 %) achieved UFC <125 % ULN (partial response) within 3–6 months of cabergoline initiation, but none of these patients experienced long-term response. Escape from response was observed in 2 patients with long-term complete response after 2 and 5 years of treatment, although one patient transiently renormalized after cabergoline dose increase. Long-term tolerability was positive; no serious adverse events were reported during treatment. No symptoms of cardiovascular dysfunction were reported and no patients presented symptomatic adrenal insufficiency.

Although the results of these small studies have shown promise for the use of cabergoline as medical therapy for CD, no large-scale, double-blind trials have been conducted. Thus, it is difficult to draw conclusions about the overall efficacy and safety of this approach.

One potential concern regarding the use of DA agonists for CD has been the association of long-term ergot derivatives with increased risk of valvular heart disease [55–57]. However, this risk was identified in studies performed in patients using DA agonists for Parkinson’s disease at doses considerably higher (often >3 mg/day) than those used in CD (generally <7 mg/week). Schade et al. [56] reported that the relative risk of valvular heart disease was 50.3 (95 % CI 6.6–381.4) in patients receiving >3 mg/day compared to 2.6 (95 % CI 0.5–12.8) in patients receiving <3 mg/day. Furthermore, a study of 78 patients receiving DA agonists (including 47 who were using cabergoline) for an average of 8 years for prolactinoma found an increased risk of aortic valve calcification and mild tricuspid regurgitation, but no increase in the risk of clinically relevant valvular heart disease compared to control patients [58]. Among patients receiving cabergoline, the mean duration of therapy was 5.2 ± 0.4 years and the mean cumulative dose was 363 ± 55 mg. There was no relation between cumulative dose of cabergoline and the presence of mild, moderate or severe valve regurgitation. Thus, the evidence suggests that valvular heart disease associated with long-term use of DA agonists is much less of a concern for patients using the doses typical of treatment regimens for CD. A summary of current prospective studies with pituitary targeted medical therapy is provided in Table 2 [40, 43, 48, 52, 53, 59].

**Table 2:** Medical treatments for Cushing’s disease evaluated in prospective clinical studies

Compound	Trial	Patients (*N*)	Treatment duration	Outcome
Pasireotide	Boscaro et al. [40]	29	15 days	5/29 patients (17 %) achieved UFC ≤ ULN; 22/29 patients (76 %) experienced reduction in mean UFC
	Colao et al. [43]	162	12 months	33/162 patients (20 %) achieved UFC ≤ ULN within 6 months without increase from randomized dose; the majority of patients experienced reduction in UFC at month 6, sustained through month 12
Cabergoline	Lila et al. [52]	18	5 months	5 patients (28 %) achieved MNSC <5 μg/dL or low-dose dexamethasone-suppressed serum cortisol <1.8 μg/dL
	Pivonello et al. [48, 53]	20	3–24 months	After 3 months, 15 patients (75 %) achieved UFC ≤ ULN or ≥25 % reduction from baseline; 8 patients (40 %) maintained UFC ≤ ULN at 24 months
Retinoic acid	Pecori Girald et al. [59]	7	6–12 months	3/7 patients (43 %) achieved UFC ≤ ULN; 5/7 patients (71 %) experienced reduction in UFC

*MNSC* midnight salivary cortisol, *UFC* urinary free cortisol, *ULN* upper limit of normal

## Other investigational therapies

### Combination somatostatin analog-dopamine agonist

Combined use of a somatostatin analog and a dopamine agonist for treatment of CD has been proposed on the basis of similar studies suggesting additive effects on growth hormone secretion from somatotroph adenomas [60]. In a study of 17 patients with CD, pasireotide monotherapy (100–250 mcg three times a day) normalized UFC levels in 5 patients, and addition of cabergoline (0.5–1.5 mg every other day) normalized UFC levels in 4 more patients. Among the remaining 8 patients, subsequent addition of ketoconazole (200 mg three times a day) normalized UFC levels in 6, leaving only 2 patients who still had elevated UFC levels. Thus, sequential therapy using these three agents shows promise but requires further studies of both efficacy and safety [61].

### Temozolomide monotherapy and in combination

Temozolomide, an oral alkylating agent typically used in chemotherapeutic treatment of astrocytoma and melanoma, has shown promise as monotherapy [62, 63], and in combination with pasireotide [64], as a treatment for aggressive pituitary adenomas and carcinomas.

To date, ~30 cases of pituitary adenoma have been treated with temozolomide (including ten ACTH-secreting adenomas). Overall reported response rate is approximately 60 % [62, 63]; however, information on temozolomide treatment of pituitary adenomas is currently confined to individual case reports and three case series [65–67]. An inverse relationship between tumoral O^6^-methylguanine-DNA methyltransferase (MGMT) immunoexpression and response to temozolomide therapy has been suggested, but not confirmed in all studies [62, 65, 67, 68].

Temozolomide may represent a viable treatment option for aggressive corticotroph adenomas refractory to surgery, radiotherapy, or other medical treatment. In tumors that responded to temozolomide, the clinical response was associated with prompt reductions in ACTH, chiasmatic compression and mass effects. Thus, it is possible to evaluate response early in the course of treatment. Targeted modulation of MGMT expression may be useful in patients who may otherwise not respond to temozolomide therapy [63].

### Retinoic acid

The retinoic acid receptor is a type II nuclear receptor involved with transcriptional regulation. In a murine corticotrope tumor cell line, retinoic acid has been shown to reduce ACTH secretion and pro-opiomelanocortin (POMC) synthesis. Further, there is evidence that, with prolonged treatment, retinoic acid induces increased caspase-3 activity and cell death in ACTH-secreting cells. It has also demonstrated inhibition of corticosterone production and cell proliferation in adrenal cortex cells [69]. Retinoic acid had demonstrated reduced ACTH secretion and prevented tumor growth in mice with implanted with tumoral corticotropes [70]. In a 6-month study in dogs with spontaneous corticotrope tumors, retinoic acid reduced cortisol excess and improved associated symptoms [71].

A recent small study showed a marked reduction in UFC levels in 5/7 patients (22–73 % of baseline values), and UFC normalization in three patients. There was no apparent pattern of decrease in plasma cortisol. Plasma ACTH decreased in the first month of treatment and then returned to pretreatment levels in responsive patients. Blood pressure, glycemia, and signs of hypercortisolism improved on treatment. Patients reported only mild adverse effects (e.g., xerophthalmia and arthralgias) [59].

### EGFR

The epidermal growth factor receptor (EGFR) family has recently been studied as a therapeutic target for CD. In cultured human and canine corticotroph tumors, gefitinib (an EGFR tyrosine kinase inhibitor) attenuated POMC expression, inhibited corticotroph tumor cell proliferation, and induced apoptosis. In murine studies, gefitinib decreased tumor size and corticosterone levels, and reversed signs of hypercortisolemia, including elevated glucose levels and excess omental fat [72]. These results indicate that inhibiting EGFR signaling may be a valuable strategy for treating CD. Further studies evaluating clinical efficacy and safety in patients with CD are needed.

### Doxazosin

The selective alpha(1)-adrenergic receptor antagonist, doxazosin has been shown to decrease plasma ACTH and inhibit proliferation in human and murine pituitary tumor cells, and may represent a potentially useful treatment option in pituitary adenomas [73]. However, there is currently no clinical experience with doxazosin, and more data are required before any such treatment strategy could be advocated.

## Other medical treatment modalities in Cushing’s disease

### Mifepristone: glucocorticoid receptor blockade

Mifepristone is a glucocorticoid receptor (GR2) antagonist, recently FDA-approved to treat hyperglycemia in adult patients with Cushing’s syndrome who have failed surgery or are not surgical candidates. Regulatory approval was based on a 24-week open-label trial studying 50 Cushing’s patients (43 with CD) and associated type 2 diabetes mellitus/impaired glucose tolerance (DM/IGT), or hypertension [74]. At the end of the treatment period, 15/25 patients (60 %) in the DM/IGT group achieved ≥25 % reductions in glucose AUC_0–120 min_. Patients also exhibited reductions in HbA_1c_ and fasting plasma glucose levels, as well as reduced body weight and waist circumference, improvements in clinical status and quality of life.

With GR2 receptor blockade, ACTH and cortisol will likely increase, potentially resulting in hypokalemia, increased blood pressure, edema, or alkalosis through activation of mineralocorticoid receptors [75]. Other potential adverse reactions include adrenal insufficiency (AI) and endometrial thickening with vaginal bleeding—both requiring close monitoring and treatment [76, 77]. As there is no biochemical marker to follow (cortisol values are not reliable), treatment efficacy and potential adrenal insufficiency must be gauged through changes in clinical signs and symptoms [77]. Mifepristone is also a potent antagonist of progesterone, thus premenopausal women must be tested for pregnancy before administration. Caution is also warranted in combination with drugs metabolized by CYP3A or CYP2C (e.g., simvastatin, cyclosporine, fentanyl, ciprofloxacin, NSAIDs, warfarin).

### Adrenal-targeted drugs

Drugs inhibiting adrenocortical steroidogenesis include ketoconazole, mitotane, etomidate and metyrapone. In general, use of these drugs requires careful clinical monitoring for adverse effects, including AI [77]. Ketoconazole has been widely used to treat CD because it inhibits several steps in adrenal steroid synthesis and reduces UFC in the majority of patients with CD [78]. However, it also inhibits androgen synthesis and is associated with liver toxicity in some patients [79]. There is little prospective information on the long-term use of adrenal-targeted agents [11, 80]; however, 1 small retrospective study showed promising results [81, 82]. Mitotane also inhibits several steps in steroidogenesis, and can be adrenolytic during long-term therapy at doses >4 g/day [77]. Because mitotane is sequestered in adipose tissue and eliminated slowly, pregnancy must be avoided for 5 years after discontinuation [77].

**Fig. 4:**
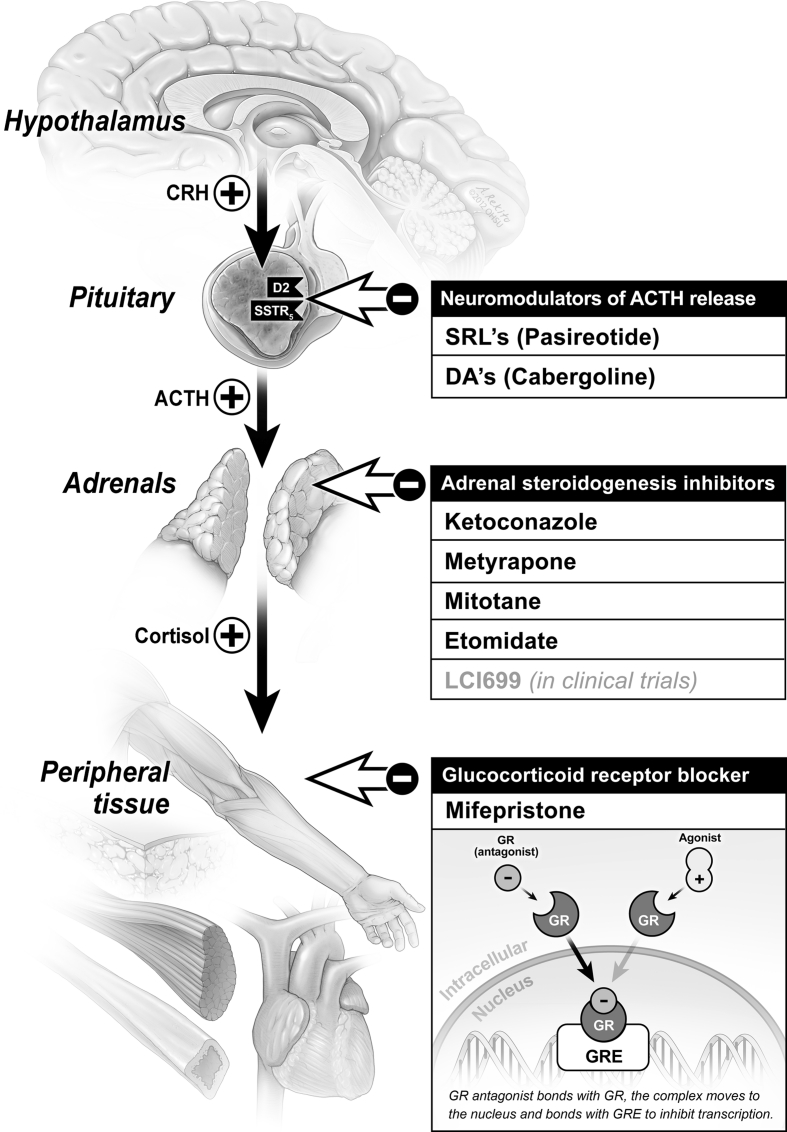
Potential targets and medical therapies in Cushing’s disease. From Fleseriu, M (2012) *Neurosurg Clin N Am* volume 23, page 657 [85]. Reproduced with permission

LCI699 is a novel inhibitor of 11β-hydroxylase (the final enzyme in the cortisol synthesis pathway) under development for several indications, including CD. In a recent proof-of-concept study in patients with CD (UFC >1.5× ULN), all participants (*N* = 12) achieved either UFC normalization or ≥50 % reduction from baseline after 70 days of treatment. While all patients experienced ≥1 AE, most were mild or moderate. Some AEs consistent with AI were reported, and resolved after dose reduction. Four patients experienced hypokalemia; all cases were managed without dose reduction, and 3 patients received oral potassium supplementation [83]. A larger-scale, 22-week expansion trial is currently underway [84]. A summary of potential therapeutic targets in CD is shown in Fig. 4 [85].

## Conclusions and future directions

Medical therapies targeting the corticotroph adenoma represent a relatively new avenue in the long term treatment of patients with CD who are unsuitable for surgery, or who fail to achieve long-term disease control post-surgery [86, 87]. Drugs targeting adrenal steroid synthesis have been in use for many years, but have not been prospectively studied and do not target the underlying pituitary tumor. A glucocorticoid receptor blocker could be a valuable asset in selected patients, but is relatively complicated to use due to absence of a biochemical marker, and there are potential adverse reactions inherent to mechanism of action.

Pasireotide, a multiligand somatostatin analog is the first pituitary-directed agent shown to effectively reduce cortisol levels and improve disease symptoms in a large, prospective, phase 3 clinical trial. Further investigation is needed, however, to clarify optimal management approaches for pasireotide-associated hyperglycemia. The dopamine agonist cabergoline has shown some promise in a small, open-label trial (and one retrospective trial), but randomized, controlled trials with this agent have not been conducted. A combination of lower doses of pasireotide with dopamine agonists or adrenal steroidogenesis inhibitors (e.g., a combination of low-dose pasireotide/cabergoline/ketoconazole) may increase the proportion of patients whose disease can be controlled while minimizing adverse effects.

Finally, further research into patterns of receptor expression in corticotroph adenomas may lead to increased understanding of the pathogenesis of these tumors, and allow development of therapies specifically tailored to individual patients following analysis of surgical pathology.
